# Study on the Optimal Leaf Area-to-Fruit Ratio of Pear Trees on the Basis of Bearing Branch Girdling and Machine Learning

**DOI:** 10.34133/plantphenomics.0233

**Published:** 2024-08-14

**Authors:** Fanhang Zhang, Qi Wang, Haitao Li, Qinyang Zhou, Zhihao Tan, Xiaochao Zu, Xin Yan, Shaoling Zhang, Seishi Ninomiya, Yue Mu, Shutian Tao

**Affiliations:** ^1^ Sanya Institute, College of Horticulture, Nanjing Agricultural University, Nanjing, Jiangsu 210095, China.; ^2^ Academy for Advanced Interdisciplinary Studies, Nanjing Agricultural University, Nanjing, Jiangsu 210095, China.; ^3^ Graduate School of Agricultural and Life Sciences, The University of Tokyo, Tokyo 188-0002, Japan.

## Abstract

The leaf area-to-fruit ratio (LAFR) is an important factor affecting fruit quality. Previous studies on LAFR have provided some recommendations for optimal values. However, these recommendations have been quite broad and lack effectiveness during the fruit thinning period. In this study, data on the LAFR and fruit quality of pears at 5 stages were collected by continuously girdling bearing branches throughout the entire fruit development process. Five different clustering algorithms, including KMeans, Agglomerative clustering, Spectral clustering, Birch, and Spectral biclustering, were employed to classify the fruit quality data. Agglomerative clustering yielded the best results when the dataset was divided into 4 clusters. The least squares method was utilized to fit the LAFR corresponding to the best quality cluster, and the optimal LAFR values for 28, 42, 63, 91, and 112 days after flowering were 12.54, 18.95, 23.79, 27.06, and 28.76 dm^2^ (the corresponding leaf-to-fruit ratio values were 19, 29, 36, 41, and 44, respectively). Furthermore, field verification experiments demonstrated that the optimal LAFR contributed to improving pear fruit quality, and a relatively high LAFR beyond the optimum value did not further increase quality. In summary, we optimized the LAFR of pear trees at different stages and confirmed the effectiveness of the optimal LAFR in improving fruit quality. Our research provides a theoretical basis for managing pear tree fruit load and achieving high-quality, clean fruit production.

## Introduction

Fruit crops hold important economic importance in agriculture and serve as essential components of the human diet [1]. In general, the market competitiveness of a fruit is determined primarily by its quality, which encompasses both its external appearance and internal quality [2]. Multiple factors influence fruit quality, with genetic material, climatic conditions, agronomic practices, and physiological mechanisms playing crucial roles [3]. Additionally, assimilate supply is a decisive factor in fruit quality [4]. The provision of assimilates is typically regulated through a synergistic interplay between plant photosynthetic capacity and the photosynthetic area [5]. Improving the photosynthetic capacity of the plant and maintaining a balance between the yield and quality of fruit poses challenges in fruit production [6]. However, modifying the source–sink relationship and carbon allocation among various plant sinks by altering the ratio of the photosynthetic area to fruit quantity [leaf area-to-fruit ratio (LAFR)] can change fruit quality and promote a balance between yield and quality [7].

In fruit production, the coordination of the source–sink relationship is accomplished primarily through fruit thinning. The leaf-to-fruit ratio (LFR; a proxLAFR) is commonly utilized as a standard for fruit thinning [8]. A high LAFR contributes positively to the development of superior fruit, whereas a low LAFR negatively affects fruit quality formation and ripening [7,9]. However, current studies have provided only a broad range of optimal LFR/LAFR values for different species or cultivars. For example, the recommended optimal LFR for *Pyrus pyrifolia* “Gold Nijisseiki” fell within the range of 35 to 50 [10]. Moreover, these recommendations were derived by comparing various fixed LFR/LAFR values, with the results typically obtained only at or near the ripe fruit stage [11,12]. As a result, these studies may overlook the dynamic changes in LAFR that occur due to the annual growth of leaves and fail to offer guidance for the critical fruit thinning period in fruit production. Furthermore, a wide range of LAFRs could have a significant effect on fruit yield and overall economic benefits. Therefore, it is imperative to address these challenges to obtain optimal LAFR and LFR values.

Girdling, a long-standing global horticultural technique involving the removal of a ring of bark, is employed to promote various beneficial outcomes, such as improved fruit maturity and quality [13]. This phenomenon can be attributed to the obstruction of the phloem, which hinders the transport of photosynthates and metabolites. Consequently, the photosynthates produced by the leaves above the girdling area are allocated primarily to support their own growth and the development of nearby fruits or other organs [13]. Previous studies have confirmed that girdling is an effective method for studying LFR. This is attributed to the ability of girdling to prevent the impact of photosynthetic products generated by other branches and leaves on the current bearing branch while simultaneously enabling efficient and accurate collection of leaf and fruit number data [4]. Therefore, continuously girdling the bearing branches throughout the entire fruit development period may offer an effective approach to studying the optimal LAFR at different stages.

Both machine learning and statistics can be used for prediction and inference, but they have different foci, predictions, or inferences [14]. Traditional statistical approaches continue to be effective in the field of biological research, operating on the premise that having sufficient data is crucial [15]. However, machine learning excels in extracting patterns from raw data without strict assumptions about the data-generating systems, making it particularly effective in complex applications [16]. Currently, machine learning methods are widely employed in plant biology, such as key transcription factor identification [17], soybean waterlogging evaluation [18], wheat nitrogen response analysis [19], and passion fruit branch detection [20]. Unsupervised learning, a primary type of machine learning, allows for pattern inference from data without the need for a dependent variable or known labels [15]. The clustering algorithm is the most extensively researched and widely utilized algorithm in the field of unsupervised learning. By calculating the distance between samples or groups, clustering algorithms can effectively gather similar samples to achieve classification. In this way, it provides a useful mechanism for pattern extraction from complex, nondependent, and unlabeled data [21]. This could offer an effective solution for handling the complex and diverse data we acquire.

Pear is an important arboreal deciduous fruit tree worldwide, and its fruit is crisp, tender, juicy, sweet, and highly nutritious [22]. According to recent data from Food and Agriculture Organization of the United Nations (FAO) (https://www.fao.org/faostat/en/#home), China’s pear cultivation accounts for 69% of the global pear cultivation area, with production exceeding 16,000,000 tons. However, the inconsistent quality of pear fruit has resulted in reduced economic benefits [23]. Therefore, enhancing pear fruit quality and maintaining suitable yields for improving economic benefits are crucial, with a reasonable pear tree load (essentially optimal LAFR) playing a key role in this process. To the best of our knowledge, cultivation research on pear trees has focused extensively on branch pruning and plant type. However, few systematic investigations have been conducted on optimizing the LAFR [8]. Therefore, it is crucial and imperative to explore the potential of pear LAFRs to achieve a harmonious balance between yield and quality and to promote sustainable, environmentally friendly production practices.

In this study, we combined girdling experiments and field verification experiments with machine learning to determine and verify the optimal LAFR at different stages for producing the best pear fruit quality while maintaining appropriate yields. In essence, our goal is to introduce a novel and effective research strategy for investigating the LAFR. By doing so, we aim to establish a solid theoretical foundation that promotes environmentally friendly and efficient cultivation practices, ultimately leading to improvements in fruit quality as well as economic benefits.

## Materials and Methods

### Experimental design and plant material

**Fig. 1.**
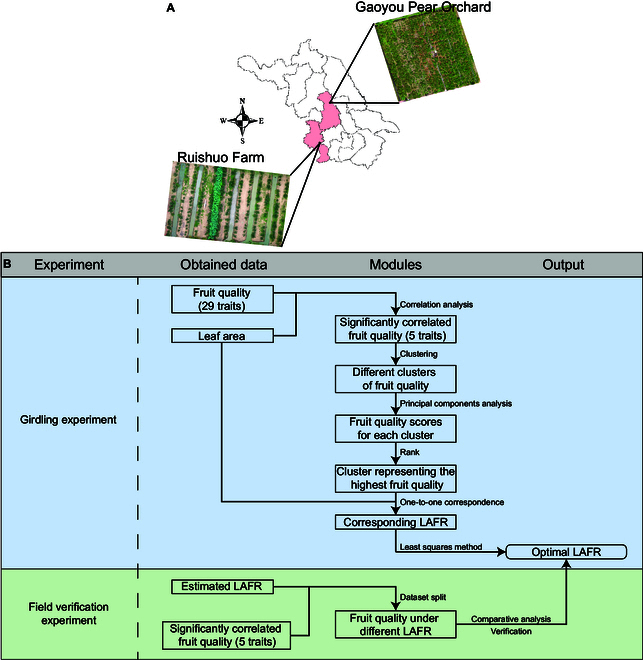
(A) Experimental units. (B) Workflow of this study. (1) In the girdling experiment, fruit quality and leaf area data at different stages were collected for each sample. Correlation analysis, clustering algorithms, principal components analysis, and the least squares method were used to determine the optimal LAFR for each stage. (2) In the field verification experiment, LAFR and fruit quality data were gathered for each pear tree. The ability of the optimal LAFR to improve fruit quality was verified by comparing fruit quality under different LAFR treatments.

The study was performed in 2021 and 2022 on a pear tree (*P. pyrifolia*) of 2 cultivars [*P. pyrifolia* “Cuiguan” (CG) and *P. pyrifolia* “Cuiyu” (CY)]. The girdling experiment was conducted in 2021 at the “Gaoyou Pear Orchard” of Nanjing Agricultural University experimental unit, located at Yangzhou, Jiangsu, China. In 2022, a field verification experiment was conducted at “Ruishuo Farm”, another experimental unit located in Nanjing, Jiangsu, China (Fig. 1A). The workflow is shown in Fig. 1B.

For the girdling experiment, 10 eight-year-old pear trees (CG) with healthy and uniform cultivation that were free from diseases and insect infections were randomly selected. Each tree received a sufficient supply of fertilizer, with 500 g of nitrogen, phosphorus, and potassium compound fertilizer applied at 14 DAF (postflowering fertilizer) and 63 DAF (fruit-enlargement fertilizer). Standardized fruit thinning was performed on the pear trees 21 days after flowering (DAF). After thinning, 20 bearing branches were chosen from each tree for girdling, with only one fruit left on each branch. The girdling width was set to 5 mm, and the wounds were wrapped with parafilm after girdling (Fig. S1). Girdling was maintained throughout the fruit development period to prevent wound healing. The leaf count and fruit size were recorded at 7, 21, 42, 70, and 91 days after girdling (DAT). These fruits were harvested at 91 DAT (112 DAF) during the fruit ripening period.

For the field validation experiment, a total of 183 seven-year-old trees were selected. Among these, 57 trees were CG, and 126 were CY. These trees were cultivated under the same and suitable conditions, with no disease or insect infection. The fertilizer supply was the same as that used in the girdling experiment. At 28 DAF, fruit thinning was conducted. At 112 DAF, fruit samples were collected, and the number of fruits and leaves on each tree was manually counted.

### LAFR determination

Following the methodology outlined by Li et al. [24], we collected mature branches with leaves at various growth stages and conducted indoor scanning via Lidar (Fig. sA and B). The automatic pipeline provided by Li et al. [24] (https://github.com/haitao971028/branch-leaf_segmentation_and_leaf_traits_extraction) was employed for the automatic segmentation of leaves and branches from the 3-dimensional (3D) point cloud. After removing leaves with missing point clouds, we subsequently used the single leaf area module of FTPCT (https://github.com/Zhang-Fanhang/FTPCT/tree/Installation-package) to reconstruct pear leaf mesh models and compute the leaf area (Fig. S2C to E). Considering the stability of the single leaf area over time (Table S1 and Fig. S2E), we utilized the average leaf area of all the leaves as a parameter for calculating the LAFR. The LAFR was calculated as follows: LAFR = leaf number × average leaf area/fruit number. Because only one pear fruit was retained on each bearing branch during the girdling experiment, the LAFR of the bearing branch could be calculated as follows: LAFR = leaf number of the bearing branch × average leaf area.

### Fruit quality determination

An electronic balance was used to determine pear fruit weight (FW). A caliper was used to determine the horizontal diameter (FHD) and longitudinal diameter (FLD) of the fruits. Fruit shape index = FLD/FHD. A pear sugar/acid meter (PAL-BX/ACID 12, ATAGO, Japan) was used to determine the soluble solids (FSSC) and acid content. A texture analyzer (CT3-4500, Brookfield, USA) was used to determine fruit firmness. The stone cell content was determined via the frozen-HCl method [25].

The extraction and determination of sugar and organic acid components followed the methods described by Wu et al. [26] and Ma et al. [27]. The sugars, including fructose, sorbitol, glucose (GLC), and sucrose, were detected via an ultraperformance liquid chromatography (UPLC) system (ACQUITY H-Class, Waters, USA). The system was equipped with a UPLC ACQUITY BEH Amide column (1.7 μm × 2.1 mm × 100 mm) and an evaporative light-scattering detector. The mobile phase consisted of 85% acetonitrile (containing 1% ammonia) and 15% ultrapure water. The flow rate was set at 0.2 ml/min, and the analysis was performed at 45°C. The injection volume was 2 μl, and the run time was 16 min. The organic acids, including oxalic acid, quinic acid, malic acid, shikimic acid, and citric acid, were detected via a UPLC system (UltiMate 3000, Thermo, USA). The system was equipped with an Acquity UPLC HSS T3 column (100 mm × 2.1 mm × 1.8 μm) and a photodiode array detector. The mobile phase consisted of 1% methanol and 99% 0.01 M KH_2_PO_4_ (pH 2.4). The flow rate was set at 0.25 ml/min, and the analysis was performed at 30°C. The injection volume was 2 μl, and the run time was 7 min.

### Statistical analysis

To determine the optimal LAFR for producing high-quality pear fruit, it is essential to identify fruit quality traits that are significantly correlated with changes in leaf area. Correlation analysis was employed to screen these traits. Five different clustering algorithms, namely, KMeans, Agglomerative clustering, Spectral clustering, Birch, and Spectral biclustering, were subsequently utilized to categorize the screened traits. This is because clustering algorithms can intelligently group similar samples and extract patterns from complex, independent, and unlabeled datasets by calculating the distance between samples [21]. Principal components analysis (PCA) was then used to identify the cluster representing the best fruit quality. Because the least squares method is widely applied to estimate the value of parameters that fit a function to a set of data and to describe the statistical properties of the estimate [28], it was used to determine the optimal LAFR (Fig. 1B).

The data were analyzed to assess significance via analysis of variance (ANOVA) and *t* test of IBM SPSS 19.0. Microsoft Office Excel 2013 was used to process the data. The Matplotlib Python package, Hiplot (https://hiplot.com.cn), and Adobe Illustrator 2020 were used to create the plots.

## Results

### Time series changes and correlation analysis of leaves and fruits

Pear fruit quality plays a crucial role in determining market competitiveness. The primary objective of studying the LAFR is to increase pear fruit quality. Hence, in this study, we selected pear trees of the same variety with identical growth conditions and developmental statues. We applied continuous girdling throughout the entire fruit development period to ensure that changes in pear fruit quality were attributable to variations in leaf area. However, achieving real-time, nondestructive, and accurate measurement of leaf area pose challenges because of the overlap and obstruction of pear tree branches and leaves during the growth period, compounded by technical limitations. Hence, following the approach of Li et al. [24], mature branches with leaves were gathered at various growth stages and scanned indoors via Lidar to extract detailed leaf area information (Fig. S2A to D). The statistical analysis revealed no significant variation in the single leaf area of the CG across the different stages (Fig. S2E). Moreover, while the maximum and minimum values of leaf area exhibited considerable disparities during the same period, the data distribution appeared to be relatively concentrated, with low standard deviation and coefficients of variation (Table S1). Therefore, the average value of the single leaf area was used to calculate the total leaf area after girdling.

**Fig. 2.**
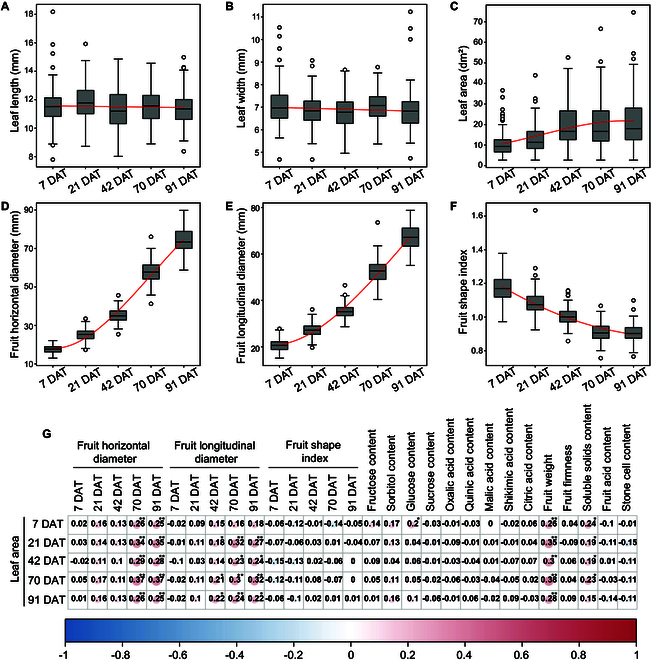
(A to F) Changes in leaf and fruit quality traits at different fruit development stages after girdling. (G) Correlation analysis between leaf area and fruit quality. Red cycles indicate positive correlations, and blue cycles indicate negative correlations. The size and shade of the circles represent the degree of correlation. Significance is indicated by “*” and “**” for *P* ≤ 0.05 and *P* ≤ 0.01, respectively.

A total of 116 fruits were harvested during pear fruit ripening. The data corresponding to these fruits regarding leaf and fruit quality were selected for analysis. The fruit quality data are shown in Table S2 and Fig. 2. The results revealed that leaf width and length remained unchanged and that the leaf area gradually increased and eventually reached a stable state (Fig. 2A to C). Additionally, FHD and FLD increased gradually, whereas the fruit shape index decreased over time (Fig. 2D to F). A correlation matrix involving leaf area and fruit quality was constructed. The results revealed a significant positive correlation between the leaf area and the FHD, FLD, FW, GLC, and FSSC of the fruits. Additionally, the FHD and FW of pear at the mature stage (91 DAT) were significantly positively correlated with leaf area at all stages. Similarly, FLD was significantly positively correlated with leaf area at stages other than 7 DAT, whereas FSSC was significantly positively correlated with leaf area at stages other than 91 DAT. However, GLC was significantly positively correlated with leaf area only at 7 DAT (Fig. 2G).

### Clustering model screening

In the aforementioned study, the fruit quality data, including FW, FHD, FLD, GLC, and FSSC data, obtained were closely linked to variations in leaf area. To determine the optimal LAFR for producing high-quality pear fruit, we needed to filter out the cluster with superior quality from the aforementioned data. However, these data exhibited complexity and independence and lacked clear differentiation. Clustering algorithms can intelligently group similar samples and extract patterns from complex, independent, and unlabeled datasets by computing the distance between samples or groups [21]. This approach offers a means for us to classify the data and identify the group exhibiting the highest fruit quality, enabling further examination of the changes in the leaf area of corresponding samples.

**Fig. 3.**
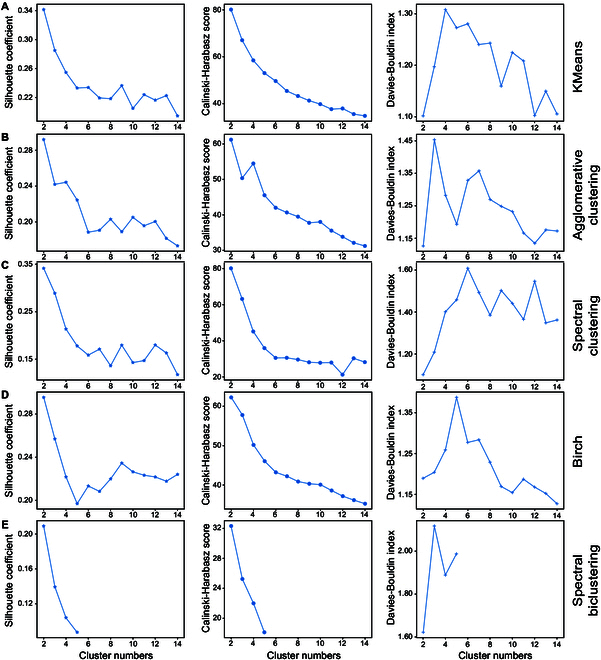
Clustering effects of different models. (A) KMeans. (B) Agglomerative clustering. (C) Spectral clustering. (D) Birch. (E) Spectral biclustering.

Five clustering algorithms, including KMeans, Agglomerative clustering, Spectral clustering, Birch, and Spectral biclustering, were applied to classify the fruit quality dataset. Each algorithm was used to classify the dataset into 2 to 14 clusters. Because the dataset is 5D, Spectral biclustering was able to classify the dataset into only 2 to 5 clusters (Fig. 3E). Three evaluation indices, the silhouette coefficient (SIC), Calinski–Harabasz score (CHS), and Davies–Bouldin index (DBI), were employed to assess the effectiveness of the clustering models. Generally, a larger SIC and CHS and a smaller DBI indicate a stronger clustering effect in the model [29–31]. The results demonstrated that the SIC and CHS of all the models exhibited a continuous decline or initial decline followed by fluctuations as the number of clusters increased. Conversely, the DBI initially increased but then subsequently decreased (Fig. 3). While all the models demonstrated improved SIC and CHS and reduced DBI when considering 2 or 3 clusters, simply dividing the dataset into 2 or 3 categories did not fulfill our requirements. Therefore, we only considered cases where the number of clusters was greater than or equal to 4. The SIC and CHS for the 5 models were highest when the number of clusters was 4 (except for the SIC of the Birch model). However, for the other 4 models, excluding Spectral biclustering, the DBI was not the lowest when the number of clusters was 4 (Fig. 3). The evaluation indices of different models under the same number of clusters were further compared. KMeans and Agglomerative clustering demonstrated the highest SIC and CHS values and relatively lower DBI values among all the models when the number of clusters was 4. Moreover, the SIC, CHS, and DBI of KMeans were slightly greater than those of Agglomerative clustering (Fig. 3). Therefore, KMeans and Agglomerative clustering were chosen to classify the dataset, and the number of clusters was 4.

### Comparison between KMeans and Agglomerative clustering

**Fig. 4.**
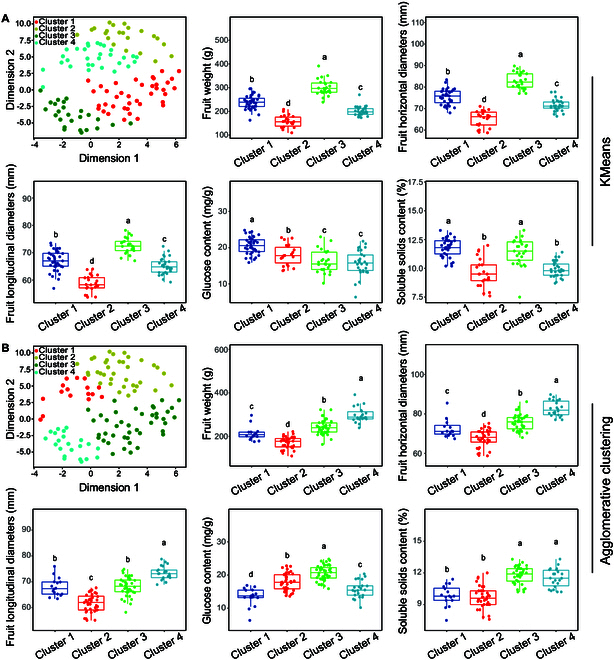
Sample distribution and 5 pear fruit quality traits after clustering by KMeans (A) and Agglomerative clustering (B). t-SNE is used for data dimensionality reduction. ANOVA was used for statistical analysis. Different lowercase letters indicate significant differences, indicated by *P* ≤ 0.05.

The above results revealed that Agglomerative clustering and the KMeans method presented similar clustering effects. Therefore, both models were used to divide the dataset into 4 clusters, and the algorithm of t-distributed stochastic neighbor embedding (t-SNE) was applied to reduce the clustered data to 2 dimensions to display the differences between clusters. The 4 clusters identified via Agglomerative clustering had more distinct boundaries than those identified via KMeans clustering. This discrepancy arises because while both KMeans and Agglomerative clustering effectively classified FW, FHD, and FLD, Agglomerative clustering also effectively classifies GLC, whereas KMeans does not. Additionally, neither of these 2 models could effectively classify FSSC (Fig. 4). Hence, using Agglomerative clustering to divide the data into 4 clusters could yield the best clustering outcome. The sample clustering results are shown in Fig. S3.

### Optimal LAFR estimation

**Fig. 5.**
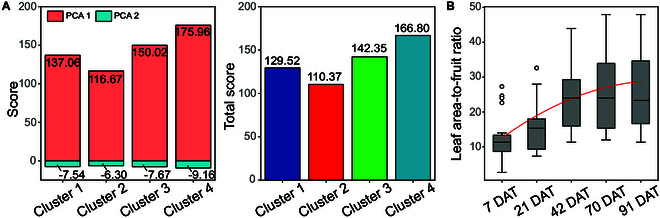
(A) Comprehensive scores of fruit quality under different clusters. (B) Change trend of the LAFR corresponding to cluster 4 and the results of the optimal value fitting.

The optimal LAFR corresponds to the highest pear fruit quality. Therefore, the corresponding cluster with the highest fruit quality was further identified via PCA. Two principal components with eigenvalues greater than one were extracted (Table S3). The results of the comprehensive evaluation revealed that cluster 4 achieved the highest scores, making it the cluster associated with the best fruit quality (Fig. 5A). This is primarily evidenced by the significantly higher values of FW, FHD, and FLD in cluster 4, along with the significantly higher FSSC than those in all clusters except cluster 3 (Fig. 4B). On this basis, the leaf area corresponding to cluster 4 was utilized to identify the LAFR. We employed the least squares method to model the leaf area data across various time periods. The intersection point of the fitted curve with the time axis was then identified as the optimal LAFR at the present time point. The results indicated that the optimal LAFR values at 7, 21, 42, 70, and 91 DAT (corresponding to 28, 42, 63, 91, and 112 DAF) were 12.54, 18.95, 23.79, 27.06, and 28.76 dm^2^, respectively, and the corresponding LFR values (calculated on the basis of the average single leaf area) were 19, 29, 36, 41, and 44, respectively (Fig. 5B).

### Comparison of fruit quality between the optimal and nonoptimal LAFRs

**Fig. 6.**
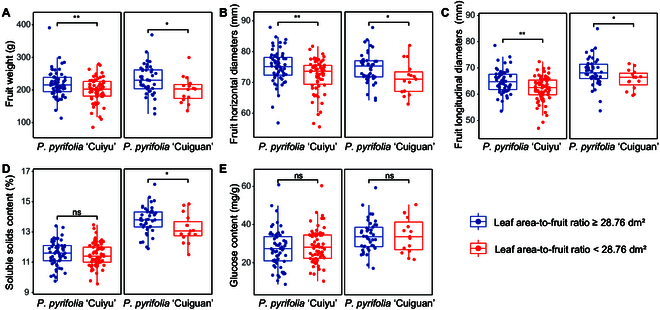
(A to E) Comparison of the fruit quality of 2 pear cultivars at optimal and nonoptimal LAFRs. A *t* test was used for statistical analysis. The significance of the differences between treatments is indicated by “*” and “**” for *P* ≤ 0.05 and *P* ≤ 0.01, respectively. “ns” indicates that the difference is not significant.

To validate the efficacy of the optimal LAFR in enhancing pear fruit quality, a total of 183 trees were utilized for field verification tests, consisting of 57 CG trees and 126 CY trees. On the basis of the LAFR values, the fruit quality data of each tree were categorized into 2 groups: LAFR < 28.76 dm^2^ and LAFR ≥ 28.76 dm^2^, corresponding to nonoptimal and optimal LAFR ranges, respectively. The comparison of the 2 groups revealed that for LAFR ≥ 28.76 dm^2^, the 2 cultivars presented significantly greater values of FW, FHD, and FLD (Fig. 6A to C). Moreover, for GLC, there were no significant differences between the 2 LAFR groups (Fig. 6E). Furthermore, at LAFR ≥ 28.76 dm^2^, the FSSC of the CG was significantly greater. However, there were no significant differences observed in another cultivar (Fig. 6D).

### Comparison of fruit quality under different LAFRs

**Fig. 7.**
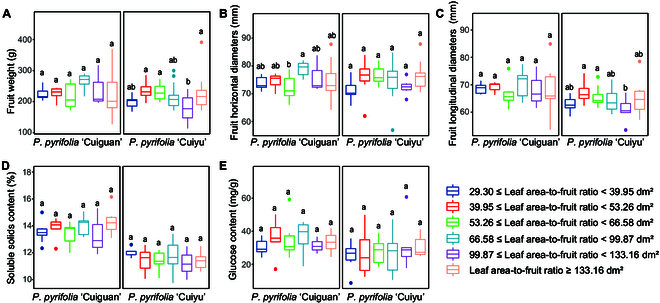
(A to E) Comparison of the fruit quality of 2 pear cultivars at 6 different LAFRs. ANOVA was used for statistical analysis. Different lowercase letters indicate significant differences, indicated by *P* ≤ 0.05.

To investigate whether the increase in LAFR could further improve fruit quality, the dataset with LAFR ≥ 28.76 dm^2^ (corresponding to LFR ≥ 44 and optimal LAFR ranges) was divided into 6 subranges on the basis of data distribution density (Fig. S4). The dataset included 42 CG trees and 60 CY trees. The fruit qualities corresponding to these 6 LAFR subranges were compared, and the results indicated that the FSSC and GLC of the 2 cultivars did not significantly differ (Fig. 7D and E). Similarly, the FW and FLD of the CG, as well as the FHD of CY, did not significantly differ among the 6 LAFR subranges (Fig. 7A to C). Additionally, although the FW and FLD of CY, as well as the FHD of CG, displayed significant differences among these 6 LAFR subranges, none of them exhibited a consistent pattern of increase with increasing LAFR. Instead, they demonstrated a fluctuating trend (Fig. 7A to C).

## Discussion

Fruit quality plays a crucial role in determining market competitiveness [2]. In China, the high yield but uneven quality of pears has resulted in a decrease in economic benefits [23]. LAFR is one of the important factors influencing fruit quality, and a high LAFR contributes positively to the development of superior fruit [9,32]. Research by Rana et al. [7] confirmed that the LFR could be calculated by girdling bearing branches. This effect was attributed to the fact that girdling had the instantaneous effect of terminating the flux of photosynthates from leaves above girdling through the phloem to elsewhere below girdling [33]. By doing so, the number of leaves required to maintain fruit growth and quality could be easily determined, and the total leaf area could also be determined [4]. Furthermore, current studies on LAFR have overlooked the fact that the number of leaves increases during the fruit development stage, leading to dynamic changes in LAFR. These methods also fail to offer guidance for the critical fruit thinning period in fruit production [10–12]. Therefore, in this study, we gathered data on pear leaf and fruit quality to determine the optimal LAFR at different stages by continuously girdling bearing branches throughout fruit development.

Photosynthates constitute the main driving force of vegetative growth and reproductive growth and are produced mainly by leaves through photosynthesis [34,35]. The size of the leaf area plays a significant role in determining the number of photosynthates [6]. Our findings revealed that the individual leaf area of a pear cultivar remained stable over time. Hence, the change in total leaf area was predominantly influenced by leaf number. In most fruit tree studies, the LFR is typically calculated by removing excess leaves [11,32]. Considering that fruit development and vegetative growth occur concurrently in many species, especially during spring [36], LFR cannot be assumed to be constant. Consequently, we collected leaf and corresponding fruit quality data at 7, 21, 42, 70, and 91 DAT (corresponding to 28, 42, 63, 91, and 112 DAF), and the leaf number was converted to the leaf area. Our results demonstrated that the continuous increase in leaf area was accompanied by a corresponding increase in fruit size, suggesting that the total leaf area positively regulated fruit quality. We further correlated the leaf area with all pear fruit quality traits via correlation analysis and found that the leaf area was significantly and positively correlated with fruit size and sugar content (including FHD, FLD, FW, GLC, and FSSC). These findings indicated that these 5 traits are key characteristics regulated by changes in leaf area.

The optimal LAFR aims to produce the best fruit quality while maintaining a reasonable yield [9–12]. In China, the economic benefits of pears are diminished because of uneven quality rather than insufficient yield [23]. Therefore, we prioritized fruit quality and needed to identify the category representing the highest quality from the data closely related to leaf area to optimize the LAFR. However, these data exhibited complexity and independence and lacked clear differentiation. A previous study has shown that clustering algorithms can intelligently group similar samples and extract patterns from complex, independent, and unlabeled datasets by computing the distance between samples or groups [21]. This provides an effective method for data processing. To analyze the dataset without any prior knowledge, 5 clustering algorithms were applied to classify the dataset [37]. The results indicated that using Agglomerative clustering to divide the data into 4 clusters yielded the best clustering outcome. The leaf area corresponding to the cluster with the best fruit quality was further examined, and the data were fitted via the least squares method, revealing that the optimal LAFRs for the 5 stages were 12.54, 18.95, 23.79, 7.06, and 28.76 dm^2^, respectively. The results converted to the optimal LFR were 19, 29, 36, 41, and 44, respectively. This finding aligns with the research conducted by Ikeda et al. [10].

To validate our conclusions, independent field verification experiments were conducted. The LAFR was calculated by recording both the leaf area converted by the leaf number and the fruit number during the fruit maturity period. Five significantly correlated fruit quality traits were also collected. Two early-maturing cultivars with similar physiological characteristics (CG and CY) were selected for these tests [38]. The results revealed that a LAFR ≥ 28.76 dm^2^ led to increased fruit size and sugar content. These findings suggest that LAFR can influence fruit quality by regulating the source–sink relationship [39]. A LAFR ≥ 28.76 dm^2^ can stimulate greater sink activity, leading to an increase in sink strength and the ability to attract carbon, ultimately increasing fruit quality [40]. The same mechanism may also be suitable for other early maturing cultivars with similar physiological characteristics. Additionally, fruit quality is the result of internal genetic programs and external environmental factors. In general, internal genetic programs determine the upper limit of fruit quality [41]. Researchers have focused on whether an increase in LAFR could further improve fruit quality. The analysis revealed that fruit quality did not improve with increasing LAFR, indicating that fruit quality had reached the limit for cultivars. These results align with those of the pear fruit quality investigation study conducted by Wu et al. [26]. This result confirmed that increasing the LAFR cannot infinitely enhance fruit quality [7]. Additionally, an increase in the LAFR can also lead to a reduction in yield [7,42]. Thus, at 112 DAF, 28.76 dm^2^ is the critical value that produces the best fruit quality while maintaining a reasonable yield, representing the optimal LAFR. LAFR values greater than or equal to 28.76 dm^2^ can maintain the highest fruit quality for pears, whereas LAFR values closer to 28.76 dm^2^ could result in increased yield.

## Conclusion

Through girdling treatment and machine learning, the optimal LAFR values for pear at different stages (28, 42, 63, 91, and 112 DAF) were determined to be 12.54, 18.95, 23.79, 27.06, and 28.76 dm^2^, and the corresponding optimal LFRs were 19, 29, 36, 41, and 44, respectively. Additionally, field verification experiments demonstrated that the optimal LAFR contributed to improving pear fruit quality, and a relatively high LAFR beyond the optimum value did not further increase quality. In conclusion, the LAFR research pipeline presented in this study is an effective strategy that can be extended to LAFR studies in other fruit trees.

## Data Availability

Data will be made available upon request.
