# Supplementary material for: Study on the Optimal Leaf Area-to-Fruit Ratio of Pear Trees on the Basis of Bearing Branch Girdling and Machine Learning
Source: Plant Phenomics. 2024 Aug 14;6:0233. doi: 10.34133/plantphenomics.0233 (PMC11322523; doi:10.34133/plantphenomics.0233)
Supplement: Supplementary 1 — Materials and Methods Figs. S1 to S4 Tables S1 to S3 [file plantphenomics.0233.f1.docx]

**Table S1** Statistics on leaf area in different periods.

| Period | Maximum value (cm^2^) | Minimum value (cm^2^) | Mean (cm^2^) | Standard deviation (cm^2^) | Coefficient of variation (%) |
| --- | --- | --- | --- | --- | --- |
| April | 90.77 | 53.48 | 65.36 | 10.22 | 15.63 |
| May | 100.64 | 54.48 | 68.69 | 12.22 | 17.80 |
| June | 90.27 | 54.15 | 65.31 | 8.85 | 13.54 |
| July | 91.67 | 53.12 | 65.28 | 10.11 | 15.48 |
| August | 99.37 | 53.61 | 68.26 | 11.60 | 16.99 |
| Total | 100.64 | 53.12 | 66.58 | 10.78 | 16.19 |

**Table S2** Statistics on fruit quality in the girdling experiment.

| Fruit quality | Maximum value | Minimum value | Mean | Standard deviation | Coefficient of variation (%) |
| --- | --- | --- | --- | --- | --- |
| Fructose content (mg/g) | 54.15 | 10.85 | 30.79 | 8.10 | 26.30 |
| Sorbitol content (mg/g) | 44.23 | 8.65 | 23.34 | 6.27 | 26.87 |
| Glucose content (mg/g) | 24.81 | 6.34 | 17.88 | 3.52 | 19.70 |
| Sucrose content (mg/g) | 5.62 | 3.52 | 4.17 | 0.56 | 13.41 |
| Oxalic acid content (mg/g) | 0.81 | 0.06 | 0.23 | 0.13 | 54.48 |
| Quinic acid content (mg/g) | 2.22 | 0.52 | 0.90 | 0.22 | 23.95 |
| Malic acid content (mg/g) | 2.13 | 0.30 | 1.32 | 0.29 | 22.14 |
| Shikimic acid content (mg/g) | 0.04 | 0.01 | 0.02 | 0.01 | 25.68 |
| Citric acid content (mg/g) | 1.28 | 0.20 | 0.60 | 0.22 | 36.13 |
| Fruit weight (g) | 391.42 | 109.52 | 226.66 | 54.88 | 24.21 |
| Fruit firmness (g) | 572.00 | 119.00 | 270.83 | 73.91 | 27.29 |
| Soluble solids content (%) | 13.31 | 7.50 | 10.82 | 1.34 | 12.36 |
| Fruit acid content (%) | 0.19 | 0.05 | 0.10 | 0.03 | 35.75 |
| Stone cell content (%) | 0.40 | 0.00 | 0.07 | 0.05 | 82.42 |

**Table S3** PCA results of different pear fruit quality clusters.

| Component | PCA 1 | PCA 2 |
| --- | --- | --- |
| Fruit weight | 0.522 | -0.058 |
| Fruit horizontal diameters | 0.521 | -0.078 |
| Fruit longitudinal diameters | 0.504 | -0.227 |
| Glucose content | -0.015 | 0.863 |
| Soluble solids content | 0.449 | 0.441 |
| Eigenvalue | 3.633 | 1.341 |
| Variance contribution rate (%) | 72.652 | 26.829 |
| Cumulative variance contribution rate (%) | 72.652 | 99.481 |


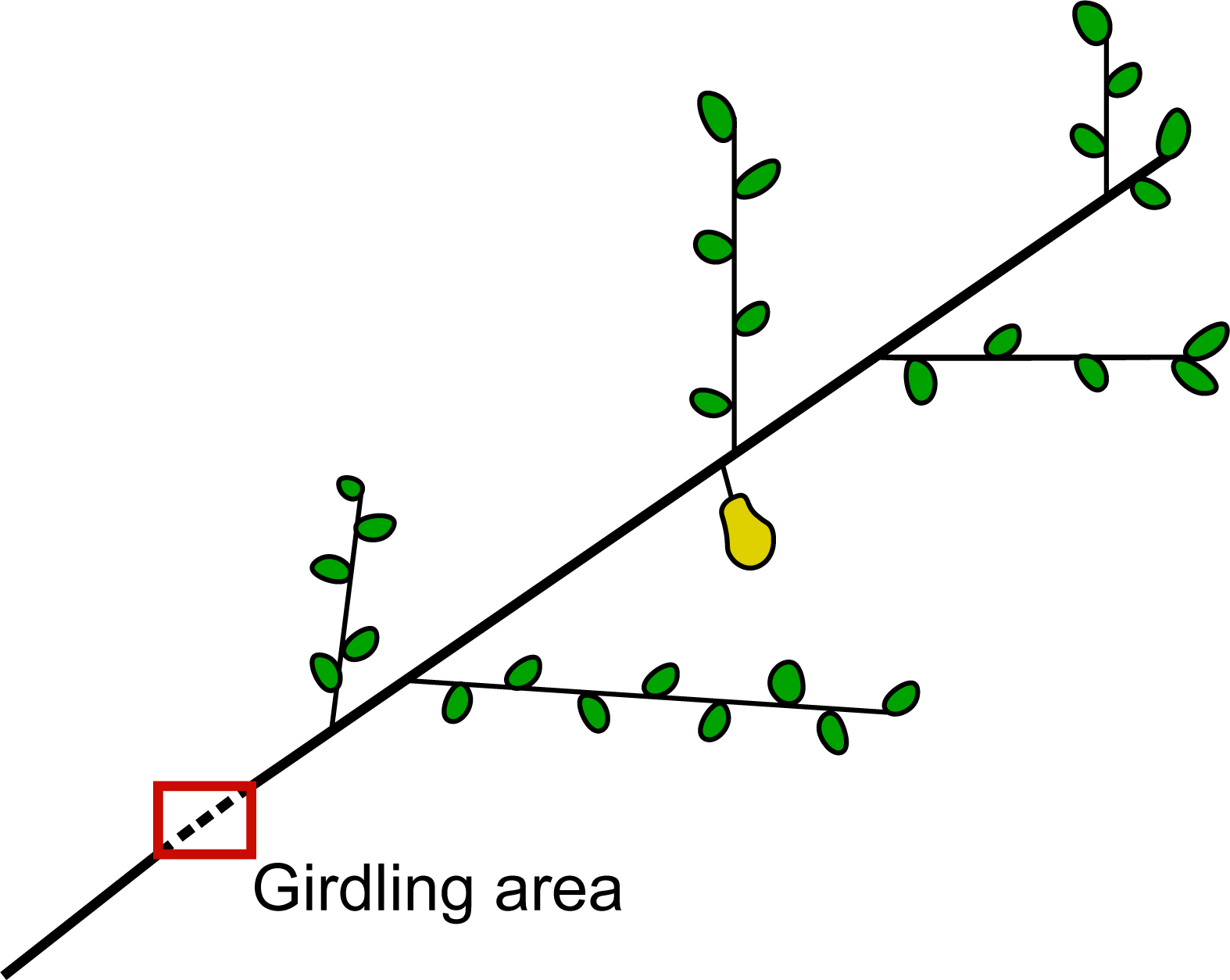


**Fig. S1** Schematic diagram of bearing branches girdling technique.


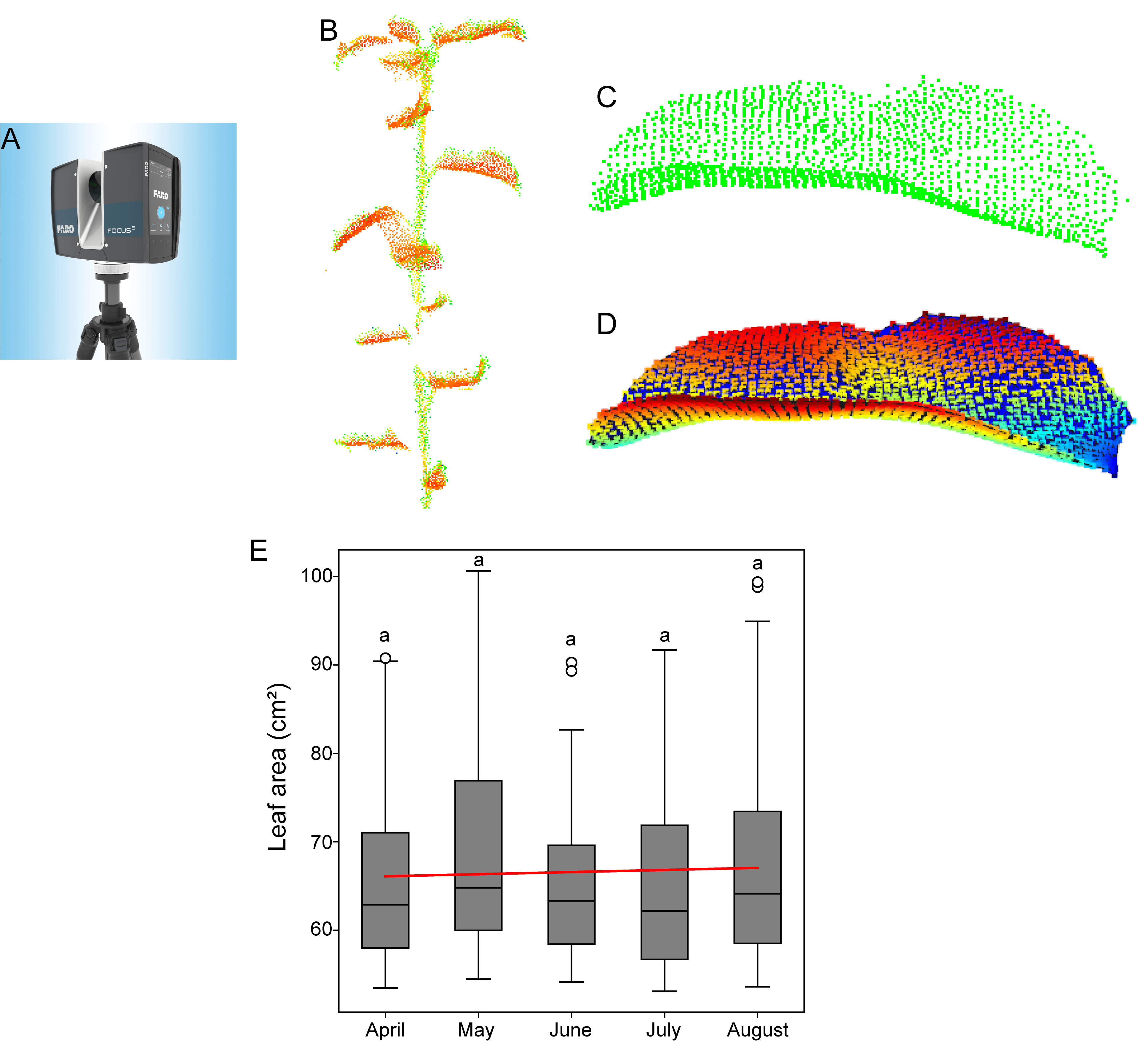


**Fig. S2** Using Lidar (A) to scan pear branches (B) for extracting 3D point cloud of leaves (C) to reconstruct mesh models of leaves by ball pivoting algorithm (D) and calculate leaf area (E). ANOVA is used for statistical analysis. The same lowercase letters indicate no significant differences


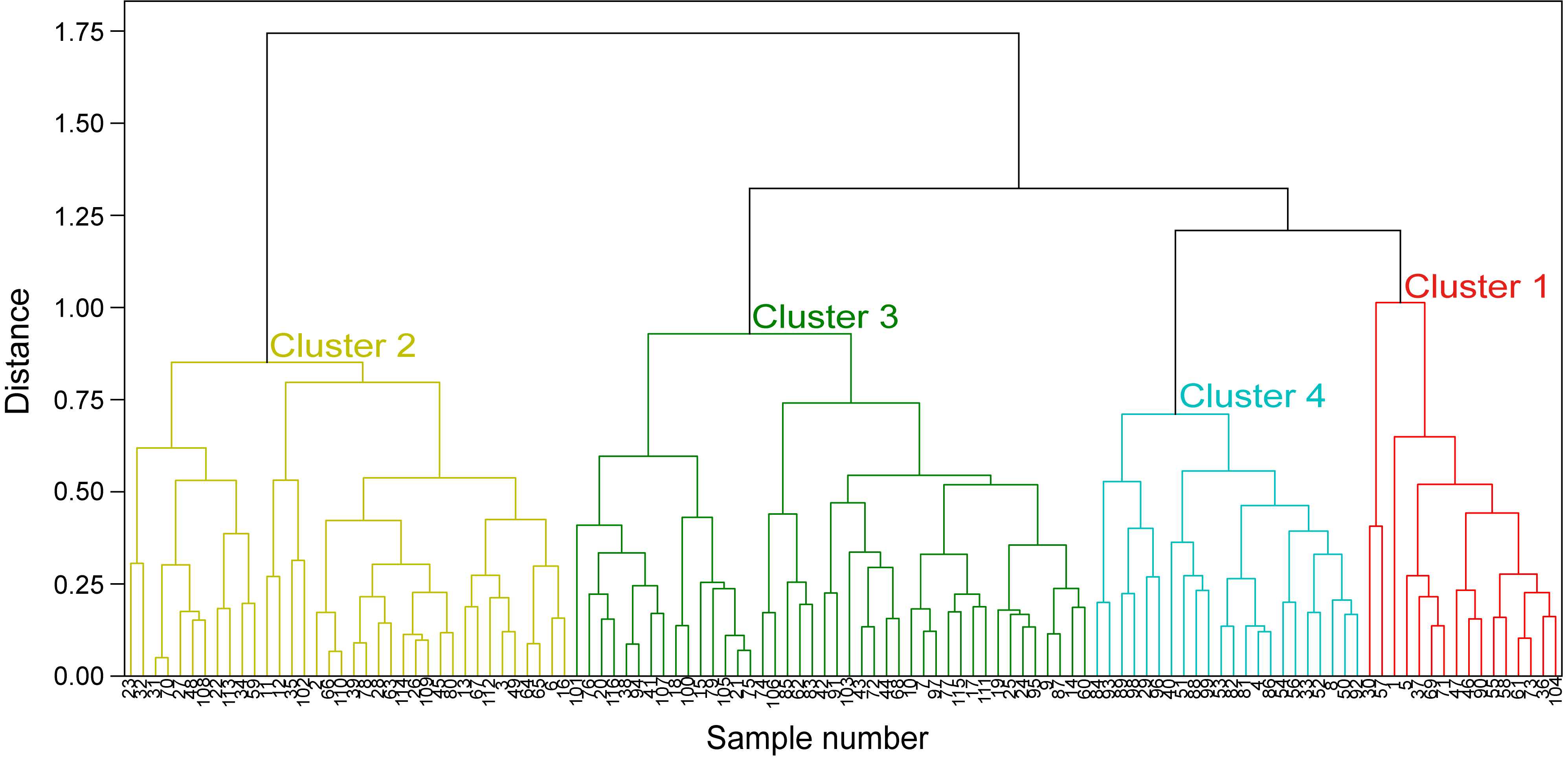


**Fig. S3** Samples distribution and hierarchical clustering tree after clustering by Agglomerative Clustering.


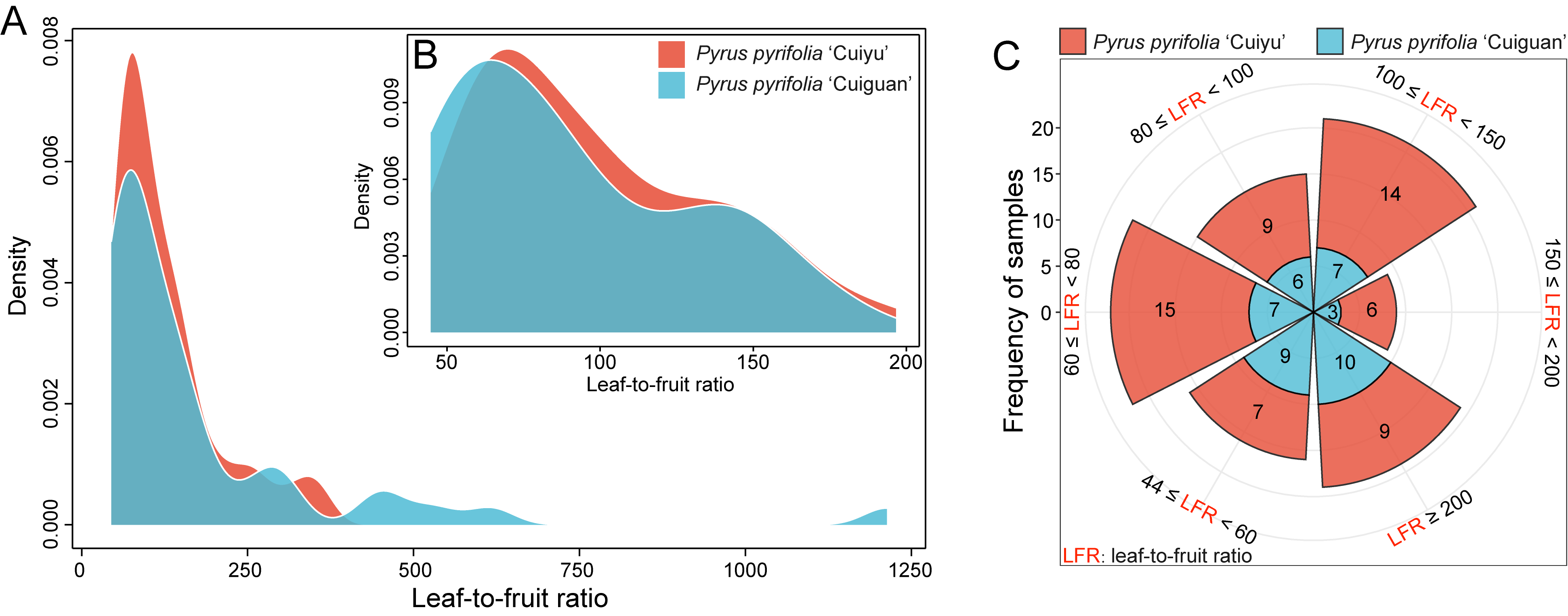


**Fig. S4** Distribution density and frequency of samples with leaf-to-fruit ratio higher than 44. (A) Distribution density of samples. (B) Distribution density of samples with leaf-to-fruit lower than 200. (C) Frequency of samples at different leaf-to-fruit ratios.
